# Extending Signaling Theory in Online Health Communities to Address Medical Information Asymmetry: Systematic Review With Narrative Synthesis

**DOI:** 10.2196/73208

**Published:** 2025-08-01

**Authors:** Shanshan Guo, Kaichao Wang, Lizhen Yang, Yuanyuan Dang

**Affiliations:** 1 Shanghai International Studies University Shanghai China; 2 South China University of Technology Guangzhou China

**Keywords:** literature review, online health community, decision-making, narrative analysis, medical asymmetry, signaling theory

## Abstract

**Background:**

In online health communities (OHCs), signaling theory has become a valuable framework for mitigating information asymmetry and shaping patient decisions. However, the literature remains fragmented, lacking an integrative understanding of how signals, signalers, receivers, and contexts interact to influence trust and engagement.

**Objective:**

This study aimed to establish a comprehensive and integrative signaling framework tailored to OHCs. It sought to clarify the core constructs of signals, categorize different signal types, and examine how signaling dynamics contribute to managing medical information asymmetry. Furthermore, this study identified key research gaps and outlined future research directions to advance the theoretical and practical application of signaling theory in digital health contexts.

**Methods:**

We conducted a systematic literature review using narrative synthesis techniques. A total of 80 peer-reviewed studies published between 2010 and 2024 were identified through 7 databases. These studies were analyzed and coded across 5 components of the signaling process: signalers, signals, receivers, signaling environments, and signaling mechanisms.

**Results:**

Five key findings emerged. First, OHC research is overwhelmingly signal centric—96% (77/80) of the studies focused on signal attributes, whereas only 3% (2/80) examined the characteristics of signalers and 14% (11/80) investigated receivers. This imbalance limits our understanding of how signals are produced and interpreted. Second, signaling mechanisms remain fragmented, with limited exploration of signal-signal or signal-context interactions. Only 31% (25/80) of the studies considered interactions between signals, and just 30% (24/80) examined contextual moderators such as uncertainty or competition. Third, environmental factors, especially environmental uncertainty and competition, play a central moderating role. Uncertain disease contexts or dense signal environments diminish signal effectiveness, particularly for affective signals. Fourth, signal classification in OHCs has become increasingly multidimensional. Signals can be systematically analyzed by their source (eg, internal vs third party), medium (eg, online vs offline), form (eg, taglike vs narrative), and affect (informative vs affective), enabling a more structured and theoretically consistent understanding. Fifth, signal interpretation is highly dependent on patient-level attributes. Patients with severe, chronic, or privacy-sensitive conditions prioritize competence or privacy signals, whereas those with limited health literacy rely more on simplified cues and affective heuristics.

**Conclusions:**

This review advances signaling theory in digital health by providing a unified framework that connects structure and context. It highlights the underexplored roles of signalers and receivers, the importance of environmental moderation, and the cognitive-emotional duality of signals. These findings offer theoretical integration and practical value for improving platform trust, patient engagement, and decision-making in OHCs.

## Introduction

### Background

Over the past decade, online health communities (OHCs) have experienced substantial growth as platforms offering remote medical consultations, physician reputation systems, and diverse information-sharing mechanisms. By facilitating real-time interactions between patients and health care providers, OHCs have significantly mitigated the information asymmetry inherent in traditional physician-patient relationships. Patients now use these platforms to evaluate physicians’ service quality through multidimensional indicators, prompting researchers to investigate how signaling theory can help explain the role of information flow in reducing uncertainty and guiding patient decisions [1-5].

Early scholarship predominantly focused on addressing information asymmetry through identifiable signal categories. These include physicians’ professional credentials (eg, academic titles, institutional affiliations, and clinical experience) [6-8], third-party certifications (eg, platform-endorsed badges and accreditation status) [9,10], and community governance norms (eg, privacy protocols and content moderation frameworks) [11,12]. Furthermore, OHCs generate behavioral metadata such as service-bundling patterns, response timeliness, and patient referral rates that function as dynamic signals to refine patients’ assessments of physician reliability [13-15]. Such signaling mechanisms collectively enhance transparency and foster trust within internet-based care ecosystems.

However, with the development of OHCs, the mechanisms of physician-patient interaction have become more complex. The role of signaling theory has expanded due to the abundance of signals, the diversity of influencing factors, and the increasing number of stakeholders [16]. Meanwhile, the theory extends the scope beyond simple effective signals to encompass the understanding of the mechanisms of information transmission within the physician-patient relationship [17], the potential impact of the physician’s image of benevolence on the patient, and so on. For instance, cumulative patient reviews and physician-patient interaction records in OHCs can be considered as physicians’ reputation signals, conveying their capabilities and kindness, and the physicians’ initiative to provide free consultation services to patients or share health knowledge for free can be regarded as the physicians’ benevolence signals, which convey kind and warm images, potentially influencing the patient’s affective commitment [3,18]. In addition, contextual factors such as physician competition, patient illness severity, and health literacy have emerged as new layers influencing signal interpretation and reception [19,20].

### The Need for an Integrated Signaling Framework

Despite the proliferation of diverse signals in OHCs, current research lacks a unified framework that systematically integrates these elements into a coherent theoretical structure. Most studies remain fragmented, focusing on either signal identification or partial classifications without fully exploring how signals interact across the signaling process.

To address this, we turned to signaling theory foundational work. Spence [21] introduced the theory in contexts of market asymmetry, where insiders (signalers) communicate unobservable qualities to outsiders (receivers). Building on this, Connelly et al [16] clarified the components of the signaling process: *the signaler*, *the signal*, *the signal receiver*, and *the environment*. Their framework has since become a cornerstone for empirical research across disciplines.

In traditional health care systems, information asymmetry has long disadvantaged patients, limiting their engagement and evaluative capacity in clinical decision-making [19,22-24]. OHCs alleviate this challenge by offering patients greater access to physician-related signals, thus expanding transparency and autonomy [1,2,15]. These include both static (eg, titles and affiliations) and dynamic (eg, responsiveness and interaction quality) indicators [2,6,25,26], enabling patients to better assess physician service quality.

While signaling theory has been widely applied in OHCs, most studies adopt a signal-centered view, describing types of signals without examining how they interact, evolve, and are interpreted in dynamic online environments [1,6,27]. Signals are rarely static or unidirectional; they emerge from complex exchanges involving multiple stakeholders (eg, physicians, platforms, and patients) and are shaped by contextual forces (eg, competition, norms, and uncertainty). Current literature has insufficiently examined the cognitive mechanisms through which patients perceive and process these signals, often assuming that they interpret all available information rationally and equally.

Moreover, emotional and affective signals (such as warmth or altruism) remain underexplored, particularly in how they interact with signaling environments such as uncertainty or normative expectations. These gaps limit the explanatory power of signaling theory in complex digital contexts.

### Study Aim

To address these gaps, this study systematically reviewed 80 peer-reviewed articles to develop a comprehensive, process-oriented signaling framework tailored to OHCs. Our proposed framework integrates signalers, signals (including source, medium, form, and affect), receivers, and environmental factors (eg, uncertainty, competition, norms, and consistency). By incorporating bounded rationality and cognitive-affective processing dynamics, the framework captures how diverse signals interact to reduce information asymmetry and shape patient decision-making.

The aim of this study was to advance a holistic understanding of signaling in OHCs by moving beyond fragmented classifications toward a dynamic, integrative framework. In doing so, we offer theoretical contributions to signaling theory and practical insights for improving patient engagement and platform design in digital health environments.

## Methods

### Search Strategy

#### Overview

We conducted a systematic review [28], a method widely recognized for its effectiveness in enhancing construct clarity and advancing theory development in signaling research [29]. Given the diversity of signaling constructs and the interdisciplinary nature of OHCs, this review required the integration of multidimensional, nonstandardized evidence across a wide range of study types and methodologies. To accommodate this complexity, we adopted a narrative synthesis approach [30] and followed a structured, multistep screening process in line with best practices for systematic reviews [31].

To identify relevant literature, we systematically searched 7 major academic databases—Web of Science, ScienceDirect, PubMed, Scopus, Wiley Online Library, Springer, and Google Scholar—covering the period from January 2010 to December 2024. We selected 2010 as the starting point for the literature search to capture the emergence of structured and research-relevant OHCs on a global scale. Around this time, major policy initiatives such as the US Health Information Technology for Economic and Clinical Health Act and the World Health Organization’s Global Strategy on Digital Health fostered the infrastructure necessary for digital health ecosystems [32,33]. Simultaneously, influential platforms such as PatientsLikeMe, HealthBoards, and WebMD expanded beyond information repositories to include physician-patient interaction, community-driven feedback, and trust signals. Interdisciplinary academic research on these platforms, particularly through signaling theory, also began to appear shortly after 2010. Therefore, this year represents a theoretically and empirically appropriate threshold for initiating our systematic review [15,34,35].

We applied a Boolean search strategy combining the core term “signal*” with 5 domain-specific terms: “online health,” “online medical,” “m-health,” “mobile health,” and “virtual health.” Searches were conducted across titles, abstracts, and keywords, resulting in an initial pool of 961 articles.

To refine the selection and minimize the inclusion of irrelevant studies, we implemented a 4-step filtering process.

#### Step 1: Identification

Following cross-database verification across the 7 databases, of the 961 articles, we removed 229 (23.8%) duplicate entries, yielding 732 (76.2%) unique records for further review.

#### Step 2: Screening

We conducted a thorough screening of the titles, abstracts, and full texts of the 732 unique records to determine their relevance to our review objectives. In this step, we applied clearly defined inclusion and exclusion criteria based on study characteristics, as recommended for systematic reviews. These criteria focused specifically on the type of publication, methodological design, topical relevance, and theoretical contribution. The applied criteria are summarized in Textbox 1.

**Inclusion and exclusion criteria.**

**Inclusion criteria**

- Study type: peer-reviewed journal articles or conference papers
- Publication date: published between January 2010 and December 2024
- Language: English
- Topical focus: online health communities (OHCs)
- Application of theory: explicit or implicit use of signaling theory or signal-related mechanisms
- Methodological design: quantitative, qualitative, or mixed methods studies
- Theoretical relevance: offering contributions to understanding of signals, signalers, or signal effects in OHCs

**Exclusion criteria**

- Study type: editorials, news articles, commentaries, and theses
- Publication date: published before 2010 or beyond the cutoff date
- Language: non-English
- Topical focus: studies on offline health care or unrelated digital platforms
- Application of theory: no conceptual or theoretical reference to signals or signaling processes
- Methodological design: nonempirical papers lacking analytical or conceptual depth
- Theoretical relevance: irrelevant or generic discussion without focus on signaling phenomena

On the basis of this screening process, of the 732 studies, we excluded 670 (91.5%), resulting in 62 (8.5%) eligible articles retained for the next phase of eligibility assessment.

#### Step 3: Eligibility

To enhance comprehensiveness, we conducted backward citation analysis of the remaining 62 articles. This yielded 17 additional peer-reviewed journal articles and 1 conference paper that met our inclusion criteria. These studies addressed signaling-related mechanisms even though the term “signal” or “signaling theory” did not appear in their titles or abstracts.

#### Step 4: Inclusion

The final sample consisted of 80 studies, which collectively provide a comprehensive overview of current theoretical and empirical understandings of signaling theory in the context of OHCs (refer to Multimedia Appendix 1 [1-10,13,14,17-20,22,24,26,27,36-95] for the full list of the included studies).

### Narrative Synthesis Approach

Given the conceptual heterogeneity and methodological diversity of the included studies, which spanned quantitative, qualitative, and mixed methods designs, this review adopted a narrative synthesis approach [96]. Narrative synthesis is particularly suited for theory-building reviews that aim to integrate nonstandardized data, identify conceptual patterns, and construct integrative frameworks across varied empirical contexts [97,98].

This approach was selected over meta-analysis or meta-synthesis due to the diversity in study designs, signaling constructs, theoretical framing, and empirical measurement. It enabled us to preserve context-specific insights while identifying cross-study regularities relevant to signaling theory in OHCs.

To ensure methodological rigor, we conducted the narrative synthesis through the following structured steps: (1) framework-guided coding based on 4 signaling components (signalers, signals, receivers, and environments) derived from the work by Connelly et al [16]; (2) extraction of analytical dimensions, including each study’s research questions, theoretical underpinnings, empirical methods, and context; (3) identification of conceptual patterns through iterative comparison and clustering of signaling mechanisms and interactions; and (4) construction of a comprehensive signaling framework integrating patterns and gaps identified across the reviewed literature.

This structured synthesis process allowed us to systematically consolidate fragmented knowledge into a cohesive theoretical structure tailored to the dynamics of OHCs.

### Quality Assessment and Coding

To ensure the relevance and conceptual rigor of the included studies, we conducted a structured quality assessment followed by a multilevel coding process. This review did not apply a formalized risk-of-bias scoring tool as is common in intervention-based meta-analyses. Instead, aligned with theory-building review practices [98,99], we adopted criteria focused on theoretical depth and topical fit.

Two coauthors independently screened and assessed the selected articles. For theoretical depth, studies were required to apply signaling theory as a central component of their conceptual framework. Articles were excluded if signaling theory was only briefly mentioned (eg, in the discussion) or used in a peripheral manner (eg, cited as a secondary explanation without elaboration). Only studies that substantively engaged with signaling theory in the context of OHCs were retained.

For topical fit, eligible studies had to focus explicitly on OHCs either as their primary empirical context or as a clearly articulated conceptual setting.

All 80 included articles met these quality standards and were treated equally in the narrative synthesis without assigning different weights based on perceived methodological rigor or publication type. This approach is consistent with the objectives of conceptual integration and theory development, where each contribution informs the construction of a comprehensive signaling framework.

Following this, we implemented a detailed coding process. Each author independently examined and coded the eligible articles based on 4 core signaling elements—signalers, signals, receivers, and signaling environments—drawing on the classification framework proposed by Connelly et al [16]. This framework served as a theoretical foundation for analyzing signaling constructs and organizing the OHC studies at a higher level of abstraction.

The coding results informed the structured narrative synthesis presented in the following sections. Articles were coded along key analytical dimensions, including research questions, theoretical grounding, empirical methods, and contextual applications. While we did not calculate interrater reliability using the Cohen κ due to the conceptual and interpretive nature of the coding, we measured coding consistency, which exceeded 95% across all dimensions.

For the few cases in which discrepancies arose, we invited an external researcher with expertise in digital health and information systems to independently review and recode the divergent sections. Final coding decisions were then reached through collaborative discussion incorporating the external expert’s feedback to achieve a high level of consensus and interpretive alignment.

This combined quality assessment and coding process ensured that only conceptually robust and theoretically relevant studies were included, laying a solid foundation for the synthesis and framework construction presented in this paper.

## Results

### Literature Search Result

**Figure 1:**
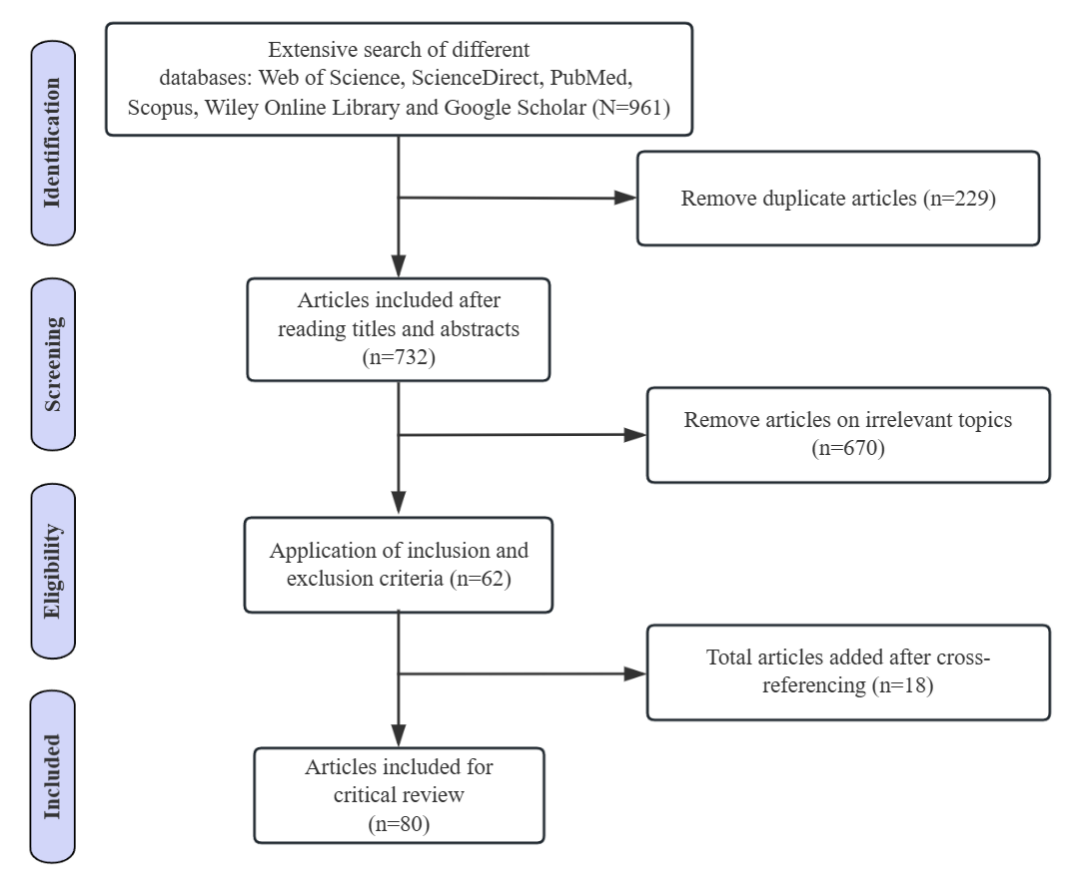
Stages of the literature search process.

The overall process of literature identification, screening, eligibility assessment, and inclusion is illustrated in Figure 1. This flowchart is adapted and modified based on the PRISMA (Preferred Reporting Items for Systematic Reviews and Meta-Analyses) 2020 guidelines [100]. A total of 80 studies were finally included in the review. The complete PRISMA checklist is provided in Multimedia Appendix 2.

### Publication Trends and Research Impact

**Figure 2:**
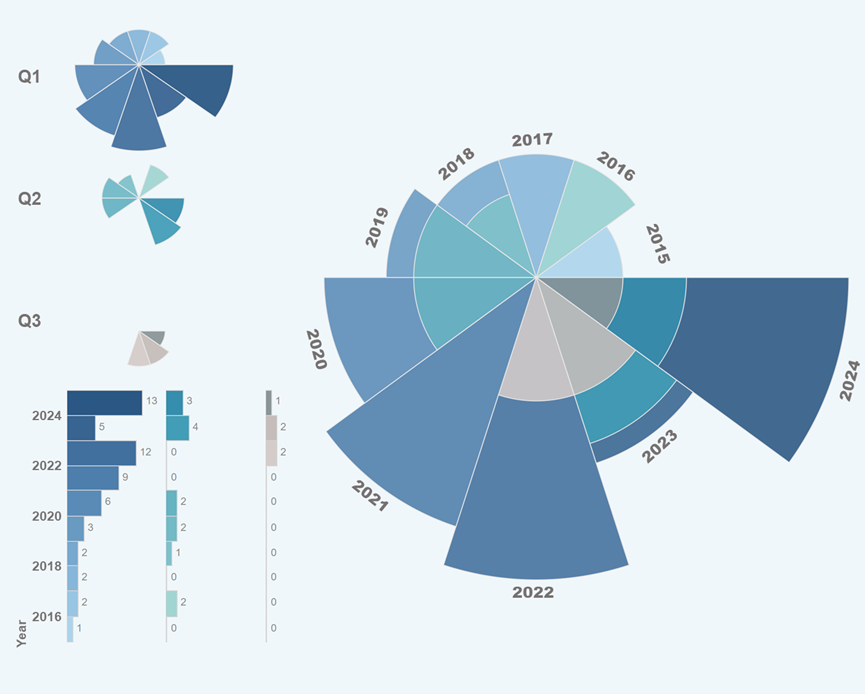
Trends in the Journal Citation Reports (JCR) category ranking by journal impact factor from 2015 to 2024. Q1 to Q3: quartile 1 to quartile 3.

The annual distribution of these articles is presented in Figure 2, illustrating the growing interest in signaling theory research within OHCs. A total of 80 studies incorporating signaling theory published over the past decade were identified. Of these 80 studies, 55 (69%) were published in journals in quartile 1 of the Science Citation Index (SCI), 14 (18%) were published in journals in quartile 2 of the SCI, and 5 (6%) were published in journals in quartile 3 of the SCI and other indexed categories, indicating an overall high quality of publications.

As shown in Figure 2, research related to signaling theory showed a consistent upward trend up to 2024. While our search covered studies from 2010 onward, the earliest eligible studies that met our inclusion criteria were published in 2015. Therefore, the trend analysis in Figure 2 reflects the period from 2015 to 2024, aligning with the actual publication timeline of the included literature. The notable number of articles published in high-impact (quartiles 1 and 2 of the SCI) journals highlights the increasing recognition and scholarly attention directed toward signaling theory. This trend underscores the growing importance of signaling theory in explaining information exchange mechanisms between physicians and patients.

In the context of rapidly expanding OHCs, signaling theory provides a crucial theoretical foundation, enabling researchers to address complex challenges related to information asymmetry and decision-making in digital health care environments.

### Signalers

#### Overview

This section identifies the primary types of signalers in OHCs and analyzes how their characteristics influence signaling behavior under conditions of information asymmetry.

Signalers, as insiders, possess privileged information about individuals, products, services, platforms, or organizations that is inaccessible to outsiders. They determine whether and how to convey this information to external audiences. In OHCs, typical signalers include physicians and platforms.

#### Physicians

In OHCs, there is a clear information asymmetry between physicians (as signalers) and patients (as receivers), placing patients in a vulnerable and uncertain position. Therefore, patients’ trust in the physician becomes a central driver of their health care decisions [36]. While signaling theory traditionally focuses on reducing asymmetry through credible cues, its deeper function in this setting is the construction of trustworthiness.

To capture how trust is built through signaling, we draw on trust theory, which identifies 3 core dimensions of perceived trustworthiness: integrity (honesty in signaling), benevolence (patient-oriented motivation), and competence (medical expertise) [101,102]. These dimensions parallel insights from source credibility theory, where perceived expertise, trustworthiness, and interpersonal appeal shape the receiver’s interpretation of a message [103]. Together, these frameworks underscore that physician signals are not only evaluated for content accuracy but also for the personal attributes they reflect that are especially salient in digital contexts lacking face-to-face reassurance.

This trust-based classification allows us to better reflect the underlying purpose of signaling in OHCs: not merely the exchange of information but also the formation of credible physician images that patients can rely on when making health-related decisions. These dimensions are particularly relevant in online environments, where traditional face-to-face cues are absent and signal credibility must be inferred from digital traces.

Integrity refers to the extent to which a physician genuinely possesses the quality suggested by the signal. Physicians with high integrity tend to emit truthful and consistent signals that accurately reflect their actual capabilities. In contrast, physicians with weaker integrity may attempt to obscure deficiencies through misleading or exaggerated signals [36,37].

Benevolence reflects a physician’s intention to prioritize patient welfare over self-interest. Benevolent physicians are more likely to engage in altruistic behaviors such as offering free consultations or publicly sharing knowledge, thereby signaling a warm and trustworthy image [38]. When patients perceive physician behavior as profit driven, their trust diminishes, and long-term engagement is jeopardized [12,27,104].

Competence captures a physician’s medical expertise and professional skill. Competent physicians signal their credibility through validated achievements such as professional titles, certifications, and accurate diagnoses. In contrast, less competent physicians may imitate credible signalers by disclosing surface-level information to influence patient judgment [37].

#### OHC Platforms

Beyond individual-level signalers such as physicians, OHC platforms function as institutional signalers, shaping the broader signaling environment through systemic cues. Grounded in institutional trust theory, platforms build trust by reducing uncertainty through structures such as organizational rules, technological safeguards, and governance mechanisms [105]. As patients lack direct interaction with platforms, perceived institutional credibility becomes essential for encouraging participation.

To further substantiate this view, we integrate online trust formation models (eg, Harrison McKnight et al [106]) that emphasize structural assurances, situational normality, and institutional reputation as key drivers of initial trust in digital contexts. These insights reinforce our classification of platform-level signals into 2 core categories: presentation quality (eg, interface usability and information accuracy) and privacy protection (eg, data encryption and anonymous services). Together, these systemic features signal platform reliability, reduce perceived risk, and foster sustained patient engagement.

Drawing from previous studies on digital platform trust [11,37,39,107,108], we categorized platform-level signals into 2 overarching dimensions.

Presentation quality includes both information quality (eg, content credibility, accuracy, and governance) and functional quality (eg, interface usability, design consistency, and user navigation). These factors shape users’ perceptions of informativeness, reliability, and ease of use, directly affecting their trust in the platform’s services [11,12].

Privacy protection refers to institutional mechanisms for safeguarding users’ sensitive data, such as encryption protocols, privacy policies, and anonymous service options. These signals are especially critical in the health care context, where patients must feel confident that their personal information will not be misused or exposed.

This classification reflects how platforms, unlike individual signalers, leverage system-level affordances to signal trustworthiness. By focusing on presentation quality and privacy protection, platforms can reduce perceived institutional risk and promote patient engagement through credible signaling structures.

### Receivers

#### Overview

Receivers, typically outsiders with limited access to professional medical knowledge, actively seek and interpret signals to reduce information asymmetry in OHCs. However, signal effectiveness is not uniform across patients. Due to variations in patients’ psychological states, motivations, and cognitive capacities, individuals often interpret the same signal in different ways. Recent studies have emphasized that the outcomes of signaling processes are partially contingent on receiver characteristics [16,19,27,40,41].

Building on this view, we categorized signal receivers in OHCs along 2 critical dimensions: illness type and cognitive ability. This classification is theoretically grounded in the principle of bounded rationality within signaling theory, which posits that individuals make decisions under cognitive and informational constraints. In the health care context, these constraints are especially salient due to the complexity of medical information and the emotional vulnerability of patients. Moreover, patients’ interpretations of signals are inherently shaped by their health-related psychological needs and information processing capacities, both of which are strongly influenced by illness characteristics and individual abilities [40-42].

#### Receivers With Different Illness Types

Patient disease attributes have been shown to significantly shape signal prioritization and health care decision-making across various OHC contexts. The severity, nature, and social sensitivity of an illness influence patients’ information needs, emotional states, and evaluation criteria when interpreting signals. For example, patients with severe illnesses (eg, tumors and cardiovascular conditions) often experience heightened anxiety and uncertainty, prompting them to prioritize signals of professional competence such as board certifications, academic titles, or hospital reputation [19,27,40-42]. Conversely, patients with milder conditions (eg, colds and gastritis) may be more responsive to relational and emotional cues such as physician friendliness and communication quality [43]. Patients managing chronic conditions (eg, diabetes and hypertension) tend to seek long-term treatment consistency and focus on informative signals related to treatment efficacy or side effects [44]. Meanwhile, patients with privacy-sensitive illnesses (eg, sexually transmitted infections and mental health issues) are particularly sensitive to both physician competence and the platform’s privacy safeguards [11,36].

#### Receivers of Different Cognitive Abilities

A patient’s ability to process health information influences the extent to which they understand and evaluate different signals. Patients with varying levels of knowledge, background, and experience exhibit diverse evaluation capacities, motivations, and expectations [9,45,109]. Patients with higher levels of disease-specific knowledge tend to rely more on direct indicators of service quality (eg, actual treatment outcomes) rather than online word-of-mouth signals [9]. In cases of complex diseases (eg, cancer and internal medicine conditions), patients must process large volumes of intricate medical information, often making signal evaluation more difficult [41]. In such cases, the quality of medical advice may be challenging to distinguish, leading to minimal differentiation between high- and low-quality signals [45]. Moreover, patients with higher health information literacy can better navigate online health information beyond basic disease knowledge. They tend to apply established evaluation criteria when assessing online health signals [109,110]. However, older adults often face cognitive and physical limitations—such as slower information processing, weaker numerical skills, and reduced ability to assess complex content—leading them to rely more on simplified signals such as images and short text descriptions [46,111-113].

The key constructs of signaling theory as applied to signalers and receivers are summarized in Table 1.

**Table 1:** Application of signaling theory constructs to signalers and receivers in online health communities (OHCs).

Perspective and contents				Example references
**Physician (signaler)**				
	**Integrity**			
		Physicians with integrity send signals consistent with their unobservable qualities.	Wang et al [40], 2020	
		Physicians with integrity are willing to disclose more detailed signals.	Gong et al [36], 2021 Liu et al [8], 2022	
	**Benevolence**			
		Benevolent physicians prioritize patient welfare over signaling costs, and their altruistic motivation strengthens patients’ long-term trust.	Yang et al [12], 2021 Chen et al [27], 2015	
	**Competence**			
		Physicians with low competence tend to disclose standardized information to imitate physicians with high competence.	Liu et al [37], 2022	
**Patient (receiver)**				
	**Illness types**			
		Disease severity—patients with high-severity illnesses (eg, tumors or heart disease) prioritize signals reflecting physicians’ professional competence (eg, certifications, titles, and hospital rankings) to alleviate pain and anxiety.	Shah et al [19], 2019 Chen et al [27], 2015 Yang et al [41], 2021 Yang et al [42], 2018 Wang et al [40], 2020	
		Chronic diseases—patients with long-term conditions (eg, hypertension and diabetes) focus on detailed health management signals such as medication efficacy and side effects.	Xia [44], 2023	
		Privacy sensitivity—patients with privacy-sensitive illnesses avoid sharing personal information online and rely more on professional competence signals (eg, credentials) rather than patient reviews.	Li et al [95] (2021)	
	**Cognitive ability**			
		Disease knowledge—in critical disease categories (eg, cancer and internal medicine), patients generally lacked the ability to discriminate medical service advice quality.	Chen and Walker [45] (2023) Li et al [95] (2021)	
		Highly knowledgeable patients prioritize actual service quality over online word of mouth.	Cao et al [9], 2017	
		Health information literacy—patients with high health information literacy rely more on established criteria. Less educated patients have poorer medical evaluation skills.	Diviani et al [109], 2015 Diviani et al [110], 2016 Chen et al [45], 2023	
		As patients age, they are more likely to have physical and cognitive impairments and worse numerical abilities. They pay more attention to less informative signals such as pictures and simplified text signals.	Heponiemi et al [111], 2023 Heponiemi et al [112], 2022 Bol et al [46], 2016 Meppelink et al [113], 2016	
**Platform (signaler)**				
	**Presentation quality**			
		Information quality regulation—the reliability of information sources, the truthfulness of information content, advertising policies, and website attributes, among other things, are crucial criteria that reflect the quality of platform information.	Guo et al [11], 2023	
		Functional quality regulation—the web page design, navigation design, visual design preferences, and page presentation style of OHCs reflect the functional quality of the platform, impacting perceived utility and patient trust.	Chang et al [39], 2022 Sbaffi and Rowley [108], 2017 Robins et al [107], 2010	
	**Privacy**			
		The protection of personal privacy by the platform promotes active signaling transmission among various parties on the platform.	Guo et al [11], 2023 Yang et al [41], 2021	

### Signals

#### Overview

In this section, we present a comprehensive classification of signal types in OHCs based on 4 core dimensions (source, medium, form, and affect) and examine their implications for patient decision-making. Signals are cues that signalers transmit to receivers to shape perceptions and behaviors. Within OHCs, researchers have identified multiple distinct signals, including physicians’ titles [42], hospital levels [4], self-representation [47], review valence [48], and patient feedback [19].

From our literature review, we observed that the key stakeholders in the signaling process within OHCs remain relatively simple—physicians, patients, and platforms—with signals flowing through physician-patient, patient-patient, and platform-patient interactions. Typically, patients are influenced by multiple signals from various participants within the information flow. However, there is a notable lack of studies that conceptually distinguished the different types of signals within OHCs. To address this gap, we classified signals based on their characteristics, as outlined in Table 2.

**Table 2:** Classification of signals in online health communities.

Signal		Source	Medium	Form	Affect	References
**Signaler-receiver—physician-patients**						
	Title	Third party	Offline	Taglike	Informative	Yang et al [42], 2018 Zhou et al [2], 2022
	Hospital level	Third party	Offline	Taglike	Informative	Li et al [49], 2019 Fan et al [13], 2023
	City	Third party	Offline	Taglike	Informative	Yang et al [41], 2021
	Experience	Third party	Offline	Taglike	Informative	Khurana et al [6], 2019 Li et al [49], 2019
	Certification	Third party	Offline	Taglike	Informative	Shah et al [19], 2019
	Service price	Internal	Online	Taglike	Informative	Yang et al [42], 2018 Wu et al [71], 2021 Wu and Lu [69], 2018
	Response to question	Internal	Online	Nontaglike	Informative and affective	Zhou et al [50], 2023 Khurana et al [6], 2019 Zhang et al [51], 2019 Zhou et al [2], 2022
	Knowledge sharing	Internal	Online	Nontaglike	Informative	Li et al [49], 2019 Ma et al [18], 2022 Zhang et al [51], 2019 Chen et al [27], 2015
	Self-representation	Internal	Online	Nontaglike	Informative and affective	Li et al [4], 2019 Ouyang et al [47], 2022 Ouyang and Wang [52], 2022
	Free consultation	Internal	Online	Nontaglike	Informative	Yan et al [38], 2022 Chen et al [27], 2015 Jiang et al [114] (2020)
	Group joining	Internal	Online	Nontaglike	Informative	Qiao et al [58], 2021 Yang et al [20] (2021)
	Bundled service	Internal	Online	Nontaglike	Informative	Yin et al [14] (2022)
	Photo or video image	Internal	Online	Nontaglike	Affective	Ouyang et al [47], 2022 Ouyang and Wang [52], 2022 Shan et al [53], 2019 Tan et al [54], 2023
	Linguistic signal	Internal	Online	Nontaglike	Informative and affective	Liu et al [17], 2023
	Sanctions	Third party	Offline	Taglike	Informative	Shah et al [19], 2019
**Signaler-receiver—patient-patient**						
	Valence of review	Internal	Online	Taglike	Informative	Wang et al [40], 2020 Chen et al [27], 2015 Wu et al [67], 2016 Lu and Rui [43], 2018
	Volume of review	Internal	Online	Taglike	Informative	Li et al [4], 2019 Huang et al [73], 2022 Shah et al [48], 2022
	Affect in review	Internal	Online	Nontaglike	Affective	Shah et al [19], 2019 Saifee et al [59], 2020
	Feedback	Internal	Online	Taglike	Informative	Yang et al [41], 2021 Yang et al [115], 2019
	Linguistic signal in post	Internal	Online	Nontaglike	Informative and affective	Chen et al [3], 2020
**Signaler-receiver—platform-patient**						
	Registration duration	Third party	Online	Taglike	Informative	Ma et al [18], 2022 Yin et al [14] (2022)
	Response speed	Third party	Online	Taglike	Informative	Yang et al [22], 2015 Guo et al [116], 2022
	Log-in behavior	Third party	Online	Taglike	Informative	Chen et al [68], 2021
	Recommendation value	Third party	Online	Taglike	Informative	Qin et al [55], 2022
	Service star badge	Third party	Online	Taglike	Informative	Cao et al [9], 2017

#### Signal Source

Signal source refers to the origin of the signal, which can be classified into internal-party signals and third-party signals. Internal-party signals are generated directly by health care providers and are not independently verified by external entities. Common examples include service price [6,19,49], response to patient inquiries [2,6,50,51], knowledge sharing [4,47], interaction records [6,49-51], and photo or video images [52-54].

Third-party signals originate from external verification sources such as medical institutions, administrative authorities, or automated platform mechanisms. These signals tend to be more objective and credible as they undergo external supervision and validation. Examples include (1) physician credentials that rely on rigorous professional evaluations by official medical associations, serving as indicators of technical competence and professional qualifications (title, hospital level, university, clinical experience, and board certification) [2,6,14,19,115,117]; (2) regulatory information that provides a verified history of malpractice issues, ensuring high information credibility (sanctions and malpractice records, which are officially recognized by national authorities) [19]; and (3) behavioral data that are automatically generated by platforms, making them immune to subjective manipulation (registration duration [14], response speed [116,118], and recommendation value [55]).

Compared to internal-party signals, third-party signals are generally more reliable as external supervision reduces the risk of misleading or false signals.

#### Signal Medium

Signal medium refers to the mode of transmission, distinguishing between offline signals and online signals.

Patients often rely on offline experiences to assess physician credibility. They are familiar with the evaluation system and organizational structure of traditional hospitals and frequently base their offline consultation decisions on physician credentials, hospital ranking, and professional certifications [4,7,56]. Because these signals align with common knowledge and established norms, patients tend to trust offline signals more readily.

Physicians generate online signals through digital interactions on OHC platforms [56,116]. These signals serve as indicators of online service quality and reflect a physician’s engagement and willingness to contribute beyond formal medical consultations [22]. Common online signals include platform-granted service badges (eg, “Service Star” recognition) [9,57]; prosocial behavior, such as knowledge sharing and voluntary contributions [38]; and participation in online health care teams [20,58].

Online signals provide a detailed view of a physician’s digital presence, showcasing their service orientation and patient engagement. In addition, certain signals exceed patient expectations, such as physicians receiving gifts or tokens of appreciation from patients, further reinforcing their commitment and care.

However, patients tend to perceive offline signals as more reliable and treat online signals as supplementary information. When offline signals strongly indicate high professional qualifications (eg, prestigious certifications and hospital affiliations), patients often rely solely on these offline credentials to make their decisions. In such cases, online signals have limited impact. When offline signals indicate lower credentials (eg, a physician holding a lower-ranking position), patients actively seek additional online signals to gain further insights [4,49]. Thus, the relative influence of online signals is context dependent, varying based on the availability and strength of offline signals.

#### Signal Form

Signal form refers to the visual representation of information transmission. Existing online platform literature distinguishes between text-only and image-text formats [119]. In addition, Hanson et al [120] explored an alternative categorization of signals, including points, badges, and labels. Inspired by these classifications, we propose 2 primary signal forms in OHCs: taglike signals and nontaglike signals.

Taglike signals are simplified, intuitive, and conspicuous indicators commonly found on search pages or physician profile pages. These signals, often marked with colors, symbols, or graphical elements, are designed to capture attention easily [121]. Platforms use these tags to summarize physicians’ past achievements, experiences, and quality characteristics, serving as a widely recognized mechanism for establishing trust [122]. Examples of taglike signals in OHCs include text-based tags (physician title [1,6], specialties [53], and work experience [2]), quantitative indicators (patient ratings [24,59], recommendation values [60], and response speed [116]), and graphic-text combinations (medals, banners, or service badges [9,57]).

Nontaglike signals are more complex and typically presented as long text, images, or videos, requiring greater cognitive effort for interpretation. These signals often demand careful analysis and deep processing as they lack immediate comparability. Examples of nontaglike signals include interaction records between physicians and patients [6,49-51], patient reviews and testimonials [3,59], health knowledge articles shared by physicians [18,104], and images and videos of physicians [52,54].

In OHCs, taglike signals reduce patients’ cognitive load, allowing for quick comparisons between different health care providers [119]. In contrast, nontaglike signals require more effortful cognitive processing. Patients may adopt different information processing strategies depending on the complexity of the signals:

- Heuristic processing—when faced with overwhelming information, patients may rely on simplified decision-making rules or cognitive shortcuts [123,124].
- Systematic processing—when motivated to make a more informed choice, patients may engage in detailed information evaluation and comprehensive analysis [124].

#### Signal Affect

Signals in OHCs can be categorized based on their functional orientation into informative signals and affective signals, each grounded in distinct psychological mechanisms but not mutually exclusive in practice.

Informative signals refer to cues that transmit factual, objective, and verifiable information about the signaler’s professional competence or service quality. These signals are designed to support systematic, cognitive processing, enabling patients to evaluate options and reduce information asymmetry. Examples include physician credentials, hospital rankings, years of experience, and response speed. While informative signals are cognitively oriented, they may also indirectly elicit emotional reassurance, particularly in high-stakes or uncertain contexts.

Affective signals refer to cues that express emotion, empathy, or relational intent through language, symbols, or visual elements. These signals influence affective heuristics and help patients navigate emotional vulnerability and build confidence in care relationships. Examples include personalized responses, empathetic language, patient appreciation notes, emoticons, and smiling physician images. However, emotionally oriented, affective signals can also shape perceptions of credibility and competence, thereby influencing cognitive evaluations [3].

These 2 types of signals are conceptually distinct yet functionally interrelated. Informative signals primarily reduce uncertainty by supporting analytical judgment, whereas affective signals foster emotional connection and trust. However, in real-world settings, many signals carry both informative and affective components, suggesting the value of studying their interaction rather than treating them as strictly separate categories.

Researchers have increasingly explored the role of affective heuristics, where emotions serve as a shortcut for judgment and decision-making, particularly in complex and uncertain medical scenarios. While cognition is crucial, affect-driven decisions often provide a faster and more intuitive response in emotionally charged health care environments.

In OHCs, informative signals primarily engage cognitive mechanisms as they directly or indirectly indicate physicians’ competence and service quality. Examples include hospital level, work experience, certification, popularity, registration duration, and response speed.

In contrast, affective signals generate emotional responses and strengthen patient engagement. Examples include personalized responses to patient inquiries [2], self-introductions and community posts [3,47], and emotionally expressive patient reviews [19,59].

Research indicates that emotional support plays a crucial role in helping patients manage health conditions [15]. Within medical consultation literature, empathy and warmth are recognized as central components of therapeutic alliances, reinforcing patient trust and satisfaction. Positive and constructive emotions not only enhance signal clarity but also increase their influence on patient choices [2].

However, most affective signals in OHCs are linguistic, meaning that they rely on verbal expressions of emotion [61]. Limited research has explored the role of nonlinguistic affective signals, such as emoticons in patient reviews, images and visual cues on platforms, and badges or other symbolic indicators of trust.

For instance, studies have found that smiles in physicians’ profile photos provide emotional reassurance and positively influence patients’ selection of health care providers [52]. Despite this, research on nonverbal affective signals in OHC decision-making remains scarce, highlighting a gap in the literature.

#### Four Key Categories of Signals

**Figure 3:**
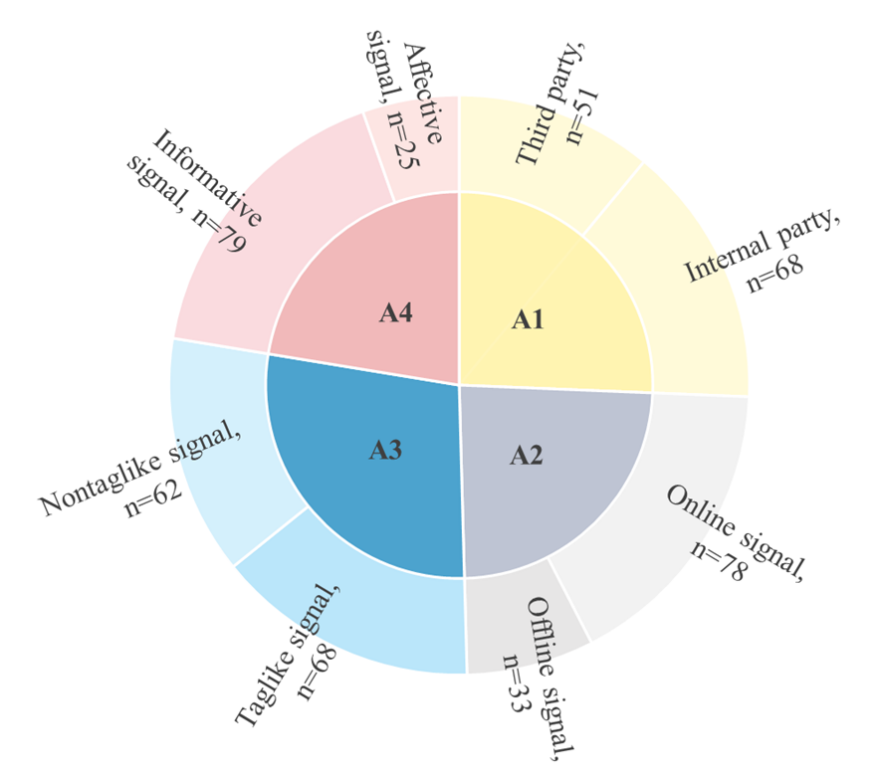
Signal classification.

In summary, we categorized signals into 4 key constructs. The signal classification map in Figure 3 enables multidimensional data visualization through Tableau’s hierarchical clustering feature (Tableau Software, LLC):

1. Signal source (A1 quadrant in Figure 3)—differentiates between third-party signals (51 instances [studies in which this type of signal was found]) and internal-party signals (68 instances).
2. Signal medium (A2 quadrant)—includes online signals (78 instances) and offline signals (33 instances).
3. Signal form (A3 quadrant)—categorizes signals into taglike signals (68 instances) and nontaglike signals (62 instances).
4. Signal affect (A4 quadrant)—comprises informative signals (79 instances) and affective signals (25 instances).

This detailed classification highlights the diversity and uniqueness of different signal types in OHCs. By structuring signals into these distinct categories, researchers can better analyze their roles and impacts within information exchange mechanisms, ultimately enhancing our understanding of how signals influence patient decision-making in digital health care environments.

### Signaling Environment

#### Overview

Signalers transmit signals within a specific environment, whereas receivers interpret these signals based on contextual factors. The signaling environment comprises both tangible elements—such as the interface design of OHCs—and intangible factors, including noise, norms, cultural influences, and expectations, all of which shape how receivers perceive and process signals.

Although some studies highlight the importance of the signaling environment in shaping signal effectiveness, there is currently no clear definition of its characteristics. To address this gap, we classified the signaling environment into 4 distinct dimensions: environmental uncertainty, environmental competition, environmental norms, and environmental consistency (as outlined in Table 3).

**Table 3:** Classification of signaling environment in online health communities (OHCs).

Construct and variable		Content	References
**Environmental uncertainty**			
	Organization reputation	A good organizational reputation has a positive effect on the physicians within the organization, decreasing patients’ uncertainty.	Liu et al [62], 2016
	Disease uncertainty	The understanding of and treatment methods for many diseases in contemporary medicine are relatively limited. This may be because the etiology of some diseases is not yet clear or because they have complex biological characteristics that increase the uncertainty of treatment.	Li et al [4], 2019 Shah et al [48], 2022 Ouyang et al [47], 2022 Ouyang and Wang [52], 2022 Shah et al [7], 2021 Wu et al [71] (2021) Shah et al [63], 2023
**Environmental competition**			
	Competition intensity	In a highly competitive environment, patients receive numerous similar signals simultaneously. The effectiveness of identical signals diminishes, whereas signals that differ from those of other signalers become more impactful.	Zhou et al [50], 2023 Fang et al [64], 2022
**Environmental norms**			
	Privacy	Patients are concerned about the platform’s regulations and its ability to safeguard their privacy.	Xue et al [65], 2023
	Consideration set size	The credibility of a signal can be enhanced by its widespread presence in the market, and signals linked to only one or a few products are insignificant. When only a few products receive ratings in a given market, patients do not perceive signals as systematic and reliable indicators of quality.	Shukla et al [60], 2021
**Environmental consistency**			
	Colleagues’ reputation	The reputation signal of a physician in an OHC is correlated with the reputation of their peers in the same environment.	Wu and Lu [67], 2016 Yin et al [66], 2022
	Content-context congruence	The congruence between the content of an answer and its contextual cues plays an indispensable role in signal receivers’ value evaluations, especially on language attributes.	Peng et al [26], 2020
	Situational abnormality	The environment deviates from a patient’s previous experience with other communities; the patient is required to put extra cognitive effort to comprehend the unusual setting.	Xue et al [65], 2023

#### Environmental Uncertainty

Environmental uncertainty refers to the various contextual factors that influence information asymmetry between signalers and receivers. In OHCs, the signaling environment provides key information about physicians, services, and institutions, helping reduce patient uncertainty.

A hospital’s reputation acts as a quality signal, influencing how patients perceive the physicians affiliated with that institution. A strong hospital reputation enhances patient confidence, reducing uncertainty in physician selection [62]. Physicians working in highly reputable hospitals signal their adherence to high medical standards, thereby positively influencing patient decision-making. When selecting physicians, patients enter specific medical submarkets that limit their choices. Differences in disease perceptions across submarkets and varying levels of medical technology development contribute to different degrees of environmental uncertainty [4,48,63].

#### Environmental Competition

Environmental competition refers to a highly dynamic setting in which multiple signalers simultaneously compete by transmitting numerous signals. In such an environment, patients are exposed to a vast number of similar signals at the same time. As a result, the effectiveness of identical signals declines, whereas signals that stand out from those of other signalers become more influential [50,64].

In the context of medical crowdsourcing services, the quality of physicians’ response content plays a crucial role in shaping how patients assess the credibility and effectiveness of medical advice [50]. However, as competition intensifies, patients receive an increasing volume of service content signals, which may lead to signal saturation and diminished signal value. The overwhelming influx of similar information makes it challenging for patients to differentiate between high- and low-quality health care providers solely based on commonly available signals.

#### Environmental Norms

Environmental norms define the order and regulations within a given environment, encompassing structured rules, organizational factors, and behavioral patterns. These norms play a crucial role in shaping how receivers interpret and respond to signals, ultimately influencing their decision-making processes.

In OHCs, patients are particularly concerned about platform regulations and privacy protection measures, which directly impact their trust in online physicians [65]. A platform’s ability to safeguard patient privacy enhances confidence in the reliability of medical consultations. Furthermore, the credibility of a signal increases when it is widely recognized in the market. Signals that are associated with only a limited number of physicians or services may be perceived as less significant [60]. For instance, when only a few physicians in a given market receive patient ratings, these signals are not perceived as systematic or reliable indicators of quality. In an OHC environment characterized by information asymmetry, patients prefer standardized signals that apply universally to facilitate comparisons and differentiate between physicians of varying quality.

#### Environmental Consistency

Environmental consistency refers to the degree of alignment between signals and the broader environmental context [26]. Some elements of signals may either align with or diverge from the surrounding environment, influencing how recipients interpret their meaning and reliability. For example, a physician’s reputation signal in an OHC is affected by the reputation of their peers within the same community [66,67]. When a physician’s colleagues are perceived to have high reputations, patients develop higher expectations for the focus physician. If the focus physician’s reputation signal does not meet these elevated expectations, the impact of their reputation signal diminishes.

Similarly, when an OHC environment significantly differs from a patient’s previous experiences with other communities, the patient must invest additional cognitive effort to understand the underlying motives behind the unusual setting [65]. This cognitive burden can lead to feelings of distrust and insecurity, ultimately reducing the patient’s willingness to rely on the platform’s information.

Another example of environmental consistency can be found in medical question-and-answer platforms, where environmental consistency refers to the alignment between an answer’s content and its contextual cues, particularly language attributes. The linguistic congruence between an answer and its surrounding context plays a pivotal role in determining the perceived value and credibility of the signal [26].

### Signal Elements and Signaling Processes

**Figure 4:**
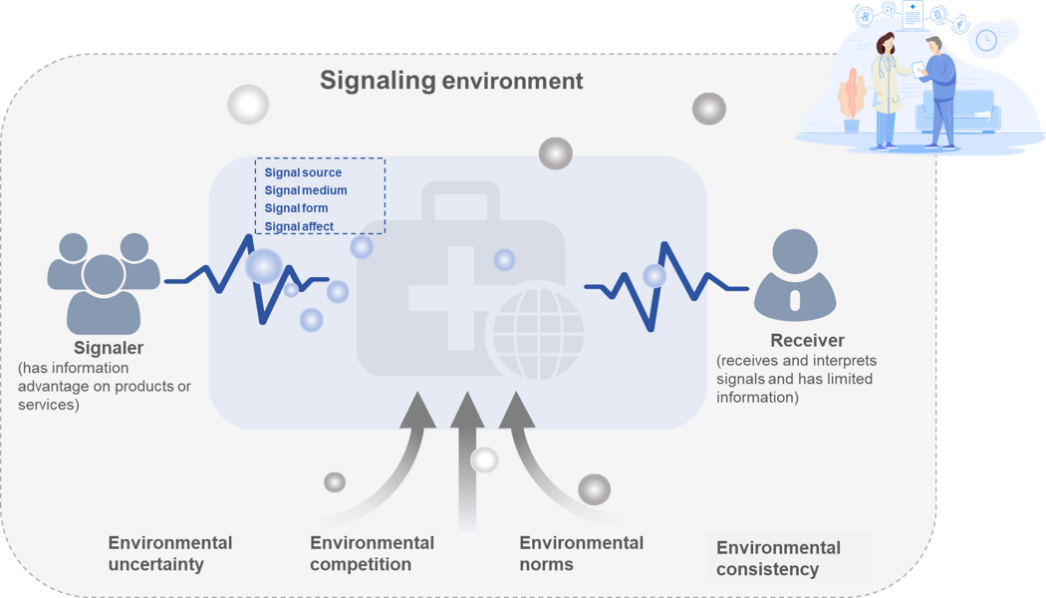
Comprehensive signaling framework diagram.

Building on the aforementioned discussions, each signal element plays an indispensable role in the signaling process, leading to the development of a comprehensive signaling framework (Figure 4). This framework illustrates the key characteristics of signal elements and their respective roles within the signaling process.

As shown in Figure 4, the four core elements of the signaling process are (1) signaler—the party possessing an information advantage regarding products or services; (2) signal—the informational cue transmitted by the signaler; (3) receiver—the party with limited access to information who receives and interprets the signal; and (4) signaling environment—the contextual setting that influences signal transmission and interpretation.

Within this framework, the signal structure is further categorized into four dimensions: (1) signal source—differentiating between internal-party and third-party signals; (2) signal medium—distinguishing between online and offline signals; (3) signal form—classifying signals as taglike or nontaglike; and (4) signal affect—defining signals as either informative or affective.

The signaling process is further shaped by environmental factors such as uncertainty, competition, norms, and consistency, all of which impact both the effectiveness of signal transmission and the receiver’s interpretation of the signal. By integrating these elements, this framework provides a comprehensive perspective on the role of signals in OHCs and their ability to mitigate information asymmetry.

### Signaling Mechanism for Navigating Medical Information Asymmetry

In recent years, research on signaling theory has grown steadily, with increasing discussions on signal transmission processes. However, despite this progress, the underlying mechanisms for navigating medical asymmetry remain somewhat ambiguous and underexplored. To address this gap, this study introduced a systematic framework to analyze the current signaling mechanisms through element coding.

For ease of description, each core signaling element is designated as follows: A represents the signaler, B represents the signal, C represents the signaling environment, and D represents the receiver.

Building on the signal classification established previously, this review categorized signals across 4 dimensions: signal source (B1), signal medium (B2), signal form (B3), and signal affect (B4).

The current research on signal transmission mechanisms primarily focuses on the individual effects of each element as well as interactions among different elements. In Multimedia Appendix 1, the signaling mechanism column is structured as follows: the plus sign (+) denotes studies investigating multiple elements simultaneously, and the asterisk (*) represents studies exploring interactions between 2 elements.

For example, in the first mechanism category, A+A*A (trustworthiness signals * online feedback) signifies a study that examines the independent effect of A (signaler characteristics) as well as the interaction between trustworthiness signals and online feedback signals. Using this coding framework, we systematically mapped the existing literature to corresponding signaling mechanisms as detailed in Multimedia Appendix 1.

**Figure 5:**
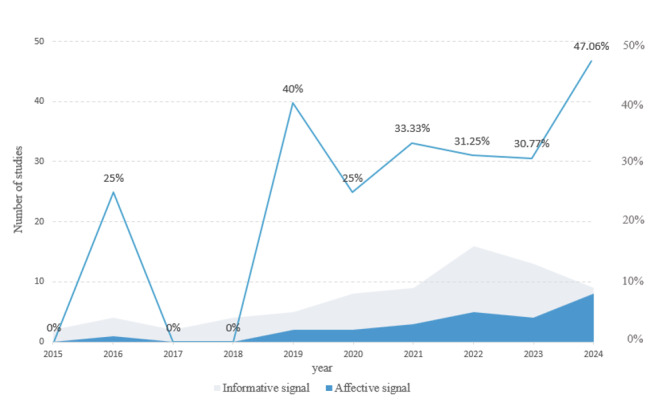
Trend in informative signals and affective signals.

To further analyze the coding results, we conducted a statistical breakdown of the 80 reviewed studies. A total of 2% (2/80) of the studies focused on signaler characteristics, whereas another 2% (2/80) explored the moderating effects of specific signaler attributes such as age, gender, and competence [37,54]. In total, 96% (77/80) of the studies examined the effects of one or more signals, with a notable increase in research on affective signals in recent years (Figure 5). A total of 31% (25/80) of the studies investigated interactions among different signals, including comparisons between online and offline signals [4,49,68], bundled services and work experience [14], and service feedback and physician popularity [63]. In total, 30% (24/80) of the studies explored the interaction between signals and environmental factors, including uncertainty, consistency, competition, and norms. A total of 14% (11/80) of the studies analyzed the influence of receiver characteristics, such as health status and cognitive abilities, on signal perception and interpretation.

This structured approach to signaling mechanisms offers a clearer understanding of how various elements interact within OHCs, providing a foundation for future research on signal transmission and medical asymmetry mitigation.

In summary, most studies (60/80, 75%) concentrated on analyzing the effects of specific signals, with a particular emphasis on informative signals. In contrast, fewer studies (13/80, 16%) explored the roles of signalers and receivers in the signaling process.

**Figure 6:**
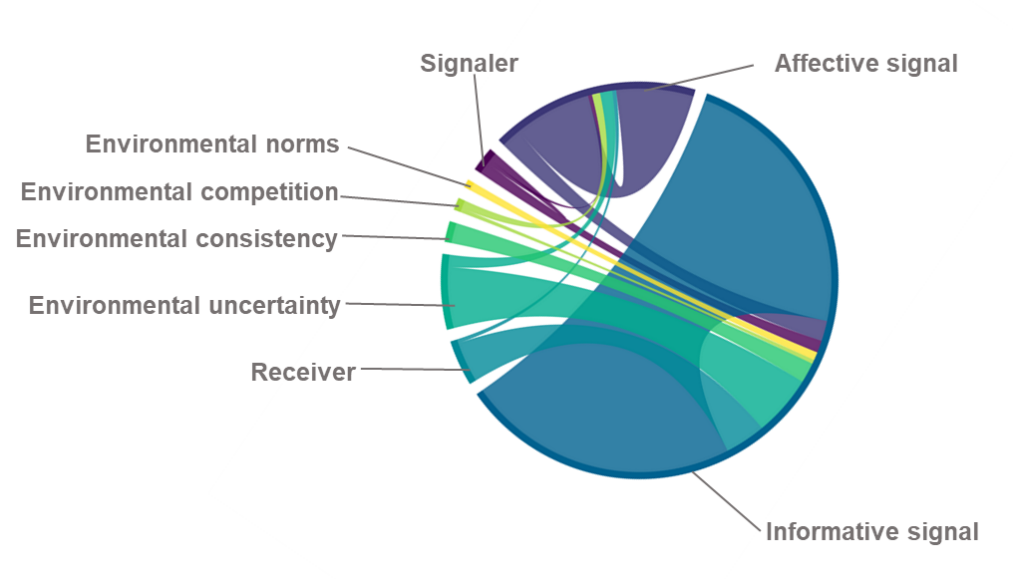
Signaling mechanism chord diagram.

To better illustrate the relationships between different signaling elements, we created a chord diagram using Matplotlib (Figure 6), which provides a visual representation of how environmental characteristics influence the transmission of informative and affective signals.

Notably, research on the interaction between affective signals and environmental factors remains relatively limited. Existing studies primarily focus on how environmental factors shape sentiment expression in language. For instance, Fang et al [64] used text analysis to quantify emotional ratings in patient reviews, applying econometric methods to examine the moderating role of environmental competition on affective signals. Their findings suggest that, in a highly competitive disease-signaling environment (ie, an environment with a greater number of physicians), emotional cues in reviews exert a stronger positive influence on patients’ selection of physicians.

Of the 24 studies that explored interactions between informative signals and the signaling environment, most examined the impact of environmental uncertainty. Specifically, 62% (15/24) of the studies reported findings related to environmental uncertainty, 25% (6/24) of the studies investigated environmental consistency, 4% (1/24) of the studies examined environmental competition, and 8% (2/24) of the studies explored environmental norms.

These findings highlight a research gap in understanding how affective signals interact with different environmental factors, suggesting an opportunity for future studies to further explore the nuanced effects of environmental dynamics on both informative and affective signaling processes.

## Discussion

### Principal Findings

This study conducted a comprehensive review to identify, organize, and interpret the diverse insights provided by existing literature on signaling theory in OHCs. Through this process, we offer an integrated overview of signalers, receivers, distinct signals, signaling environments, and signaling mechanisms. On the basis of the results, 5 key findings emerged.

First, there was a notable imbalance in the research focus. While 96% (77/80) of the studies concentrated on signal attributes, particularly informative signals such as titles, credentials, and response metrics, relatively few studies investigated the characteristics of signalers (2/80, 3%) or receivers (11/80, 14%). This indicates an overly signal-centered research paradigm and suggests a need to better integrate the roles of senders and interpreters in the signaling process.

Second, signaling mechanisms remain fragmented, with limited attention to how multiple elements interact. While most studies analyzed single-signal effects, relatively few explored how signals interact with one another (25/80, 31%) or with environmental conditions (24/80, 30%). Among those that did, informative signals dominated, and studies examining affective signals such as empathy in physician responses or emotional tones in patient reviews were both fewer (25/80, 31%) and relatively undertheorized.

Third, environmental factors play a key moderating role, especially environmental uncertainty and environmental competition. Our coding shows that 62% (15/24) of the studies on environmental interactions reported findings related to uncertainty (eg, disease ambiguity and technological opacity), whereas signal saturation under competitive conditions can reduce the effectiveness of otherwise strong cues such as badges or recommendations. Other environmental dimensions such as consistency and normative expectations remain underexplored, particularly in how they shape the credibility or visibility of affective signals.

Fourth, signal classification in OHCs is multidimensional and increasingly sophisticated. Our review identified key dimensions—source (internal vs third party), medium (online vs offline), form (taglike vs nontaglike), and affect (informative vs affective)—that offer a structured lens to analyze diverse signals. However, patients often rely more heavily on offline or third party–verified signals, especially when cognitive constraints or information overload limit their ability to process online cues.

Fifth, despite increasing signal complexity, signal interpretation remains highly contingent on patient attributes such as illness type and cognitive ability. Patients with severe, chronic, or privacy-sensitive illnesses prioritize different types of signals (eg, competence vs privacy). Similarly, older adults or those with low health literacy rely more on heuristic processing and simplified visual signals. These findings highlight the bounded rationality of receivers and emphasize the importance of designing signals that match users’ psychological and informational capacities.

Building on the findings of this review, we propose several key research directions that warrant further exploration regarding OHCs.

### Embracing a Holistic Perspective

#### Overview

An emerging research trend involves applying a holistic perspective to study signaling processes within OHCs [4,41,64]. While scholars have implicitly incorporated holistic thinking into empirical research, particularly by exploring environmental factors, receivers, and senders, most studies only acknowledge that signal effectiveness varies under different conditions but lack in-depth theoretical investigations into these variations.

For example, research on signaling environments frequently examines disease risk yet fails to conceptualize disease itself as an environmental signaling factor [19,61]. Similarly, while studies acknowledge that contextual conditions shape signal effectiveness [26], they often lack a structured framework for understanding how signals are generated, assigned meaning, and interpreted as part of an interconnected process.

To address these gaps, we propose that future research should approach signal transmission as a holistic process in which signals are not only transmitted but also endowed with meaning through interpretation. On the basis of this perspective, we outline key research avenues that remain underexplored within OHCs and warrant further investigation.

#### Dynamic Signal Interaction and Interpretation

Future research should conceptualize signal transmission in OHCs as a holistic, indivisible process in which signals are generated, endowed with meaning, and dynamically interpreted. This perspective aligns with emerging research trends emphasizing the interactive nature of signals. For example, Zhou et al [2] argue that simultaneous interactions between signals on online health platforms can affect one another, leading to complementary or substitution effects, with boundary conditions shaping signal effectiveness. This interaction highlights the context-dependent nature of health-related signal interpretation by community members.

The dynamic nature of signal interpretation in OHCs underscores the need for a more integrated analytical framework that accounts for how various signals, such as user comments, ratings, physician endorsements, and trust signals, interact and influence one another. Understanding these interactions can lead to a deeper appreciation of the complexities involved in health communication within these platforms, enabling researchers to refine theories and models that reflect the real-world dynamics of online interactions. By considering the relationships between different signals and their boundary conditions, scholars can identify new variables and moderating factors that influence signal effectiveness and member interpretation, ultimately leading to a more predictive and nuanced understanding of patient decision-making.

Adopting a holistic perspective on signal transmission and interpretation in OHCs can significantly advance the study of health communication. Examining the dynamic interactions between signals and the role of the signaling environment allows for the development of more sophisticated models that better capture the complexity of health-related decision-making in digital communities. This approach contributes to a deeper understanding of online health communication processes and holds practical implications for enhancing the effectiveness of OHCs in guiding patients toward informed and beneficial health choices.

#### Environment Dynamics and Optimal Signal Timings

Fang et al [64] referenced the framework by Connelly et al [16] to introduce the concept of the signal environment, emphasizing that both signal effectiveness and the receiver’s ability to accurately evaluate signals are shaped by environmental factors such as market intensity. In the context of OHCs, the signal environment extends beyond traditional market conditions to include environmental uncertainty, environmental norms, environmental competition, and environmental consistency. These factors collectively influence how signals are perceived, interpreted, and acted upon by patients and other community members.

Beyond environmental dynamics, research suggests that signals possess time-varying properties, making the determination of optimal timing a crucial factor in effective communication [1,125]. However, our understanding remains limited regarding how signalers identify the most effective timing for signal transmission and how receivers process and respond to signals over time. The dynamic nature of health communication in OHCs requires a more systematic exploration of these aspects.

To advance research on signal dynamics and optimal timing in OHCs, future studies should examine the mechanisms through which signalers determine the most effective timing for delivering health-related messages and analyze how receivers interpret and act on signals over different time frames. In addition, understanding the impact of timing on user trust and engagement is essential as well-timed signals can enhance trust, encourage participation, and strengthen patient-physician interactions, whereas poorly timed signals may contribute to misinformation and decreased trust [126].

Developing adaptive and dynamic signaling strategies that respond to the evolving conditions within OHCs will be critical in ensuring effective communication and improved health outcomes. By addressing these gaps, researchers can contribute to a more comprehensive understanding of optimizing signal timing in OHCs, ultimately leading to more impactful health interventions and better patient engagement.

#### Research Imbalance on Signal Transmission

While our review identified significant patterns in signal transmission, several critical research gaps remain. The most pressing issue is the current imbalance in research focus, where signalers and receivers are less frequently examined compared to signals and the signaling environment. This disproportionate attention has led to a lack of clarity in understanding how signalers transmit signals and how receivers interpret them within OHCs.

One possible reason for the scarcity of direct studies on signalers is the inherent opacity surrounding their characteristics, making it difficult for researchers to observe and analyze them directly [16,21]. Signalers have incentives to conceal their true traits, sometimes engaging in mimicry or deception to appear indistinguishable from other products and services [2]. As a result, while signals can provide clues about signaler quality and credibility, the relationship between signals and signalers remains ambiguous. For instance, some signalers intentionally use standardized signals to obscure low actual quality, whereas others fail to fully leverage signal transmission, leading to underestimated effectiveness in showcasing their true attributes [37]. Different types of signalers use varying signaling strategies and transmission methods, yet it remains unclear how these differences influence receivers’ trust and decision-making.

Similarly, current research lacks sufficient understanding of receivers, often blurring the distinction between receivers and the signaling environment. For example, in the context of disease risk, some studies interpret it as a signal received by individuals [19,61], whereas others categorize it as a part of the signaling environment [4,41,64]. The key distinction between receivers and the environment lies in the fact that the signaling environment affects all signals within it, whereas individuals within the same environment may interpret signals differently based on health literacy, psychological state, and personal biases. In the case of high-risk diseases, all stakeholders face similar informational constraints due to medical technology limitations. However, individual patients may process and respond to the same signals in vastly different ways, leading to unpredictable biases in decision-making.

Thus, our review highlights 2 critical gaps in signal transmission research: first, the challenge of accurately capturing signaler characteristics due to strategic concealment and signal manipulation and, second, the tendency to conflate receivers with the signaling environment, leading to unclear boundaries in analytical frameworks. Addressing these gaps is crucial for achieving a comprehensive understanding of signal dynamics in OHCs. By clarifying the relationship between signalers and signals and by differentiating receivers from the environment, future research can develop more precise models to explain how individuals navigate health-related signals in digital health care ecosystems.

#### Incorporating Psychological Mechanisms Into Signal Cognition

This study highlights the importance of incorporating psychological mechanisms into the understanding of signal cognition. Traditional signaling theory often assumes that each signal is received and processed equally, with its effectiveness determined by its inherent strength or credibility [2,16]. However, given the overwhelming volume of information generated daily on online platforms, individuals cannot process all signals equally [125], challenging this traditional assumption. Developing a comprehensive framework that integrates psychological and cognitive mechanisms into signal reception and interpretation can enhance our understanding of how signals are generated, used, and responded to, thereby complementing and expanding signaling theory.

Future research should further investigate the relationship between heuristics and signal reception. Heuristic thinking simplifies decision-making by applying experience-based strategies and cognitive shortcuts, enabling individuals to make quick judgments while reducing cognitive load [127]. In rapid and heuristic processing, emotional states often serve as informational cues, allowing individuals to form evaluative judgments without engaging in deep analytical reasoning [128,129]. Our review observed a growing presence of affective signals in OHCs, which may signify the emergence of a new signaling subcategory.

However, the interaction between affective signals and the environment remains uncertain. For instance, environmental uncertainty may lead individuals to rely more heavily on heuristic cues, increasing the influence of affective signals on decision-making [130]. Conversely, environmental consistency may reinforce reliance on emotional states as a basis for judgment, further simplifying cognitive processing. Dedicated research is required to examine how affective signals interact with environmental factors and determine whether their influence on decision-making is ultimately beneficial or detrimental. By incorporating psychological mechanisms into signal cognition, future studies can offer a more nuanced perspective on signaling theory, particularly within digital health ecosystems.

### Practical Implications for OHC Design and Management

In addition to its theoretical contributions, this study offers several practical implications for the design and management of OHCs. By leveraging the proposed multidimensional signaling framework, platform developers and health care providers can improve both the credibility of digital platforms and the engagement of their users.

First, signal hierarchy and visibility should be optimized. Platforms can prioritize the display of third party–verified, high-credibility informative signals (eg, physician certifications and hospital affiliations) on physicians’ profile pages. Affective signals such as personalized greetings, thank-you badges, or empathetic language can be strategically placed to build emotional resonance without overwhelming cognitive processing.

Second, the contextual relevance and timing of signals should be calibrated. In high-uncertainty environments (eg, mental health forums or rare disease sections), OHCs can amplify affective cues to reduce anxiety, whereas in chronic care or high-risk illness contexts, informative signals should be emphasized. This requires adaptive interface designs that respond dynamically to users’ psychological needs and informational preferences.

Third, user segmentation strategies can be designed around receiver characteristics. For example, older adults or users with lower health literacy may benefit from simplified visual signals (eg, badges and icons) and personalized recommendations, whereas medically literate users may prefer access to detailed data and structured reviews. Tailoring signal formats to patient profiles can enhance user satisfaction and trust.

Finally, platform governance mechanisms such as signal authenticity verification, dynamic feedback systems, and privacy guarantees (eg, encrypted interfaces and anonymous posting options) are essential to building and maintaining long-term trust within OHCs.

By embedding signaling theory into the platform’s design logic, OHCs can better address information asymmetry, foster meaningful interaction, and support more informed health decision-making.

## Appendix

Multimedia Appendix 1 Summary of signaling mechanisms.

Multimedia Appendix 2 PRISMA (Preferred Reporting Items for Systematic Reviews and Meta-Analyses) checklist.

## Abbreviations

OHC: online health community
PRISMA: Preferred Reporting Items for Systematic Reviews and Meta-Analyses
SCI: Science Citation Index

## Data Availability

The datasets generated or analyzed during this study are available in Google Drive [131].
