# Supplementary material for: Extending Signaling Theory in Online Health Communities to Address Medical Information Asymmetry: Systematic Review With Narrative Synthesis
Source: J Med Internet Res. 2025 Aug 1;27:e73208. doi: 10.2196/73208 (PMC12357127; doi:10.2196/73208)
Supplement: Multimedia Appendix 1 [file jmir_v27i1e73208_app1.docx]

**Appendix 1**

Table Appendix 1. Summary of signaling mechanisms

| References | Signaler | Signal Source | Signal Medium | Signal Form | Signaling Affect | Environment | Receiver | Signaling Mechanism |
| --- | --- | --- | --- | --- | --- | --- | --- | --- |
|  | A | B1 | B2 | B3 | B4 | C | D |  |
| Chen et al [27], 2015 | -- | Third Party, Internal party | Offline Signal, Online Signal | Tag-like Signal, Non-Tag-like Signal | Informative Signal | -- | -- | B+B*B |
| Yang et al [22], 2015 | -- | Third Party, Internal party | Online Signal | Tag-like Signal | Informative Signal | -- | -- | B+B*B |
| Wu and Lu [67], 2016 | -- | Internal Party | Online Signal | Tag-like Signal | Informative Signal | Environmental consistency | -- | B+B*C |
| Liu et al [62], 2016 | -- | Third party | Offline Signal Online Signal | Tag-like Signal | Informative Signal | Environmental uncertainty | -- | B+B*C |
| Li et al [56], 2016 | -- | Internal Party | Online Signal | Tag-like Signal, Non-Tag-like Signal | Informative Signal | -- | -- | B |
| Bol et al [46], 2016 | -- | Third Party | Online Signal | Non-Tag-like Signal | Informative Signal, Affective Signal | -- | Health information literacy | B+B*D |
| Cao et al [9], 2017 | -- | Third Party, Internal Party | Online Signal | Tag-like Signal, Non-Tag-like Signal | Informative Signal | Environmental uncertainty | Disease knowledge | B+B*D+B*C |
| Hampshire et al [10], 2017 | -- | Third Party, Internal Party | Offline Signal | Tag-like Signal, Non-Tag-like Signal | Informative Signal | -- | Disease knowledge | B+B*D |
| Lu and Rui [43], 2018 | -- | Internal Party | Online Signal | Tag-like Signal | Informative Signal | -- | -- | B |
| Li et al [4], 2019 | -- | Third Party, Internal Party | Offline Signal, Online Signal | Tag-like Signal, Non-Tag-like Signal | Informative Signal | Environmental uncertainty | -- | B+B*C |
| Wu and Lu [69], 2018 | -- | Internal party | Online Signal | Tag-like Signal, Non-Tag-like Signal | Informative Signal | -- | -- | B |
| Yang et al [42], 2018 | -- | Third Party, Internal Party | Offline Signal, Online Signal | Tag-like Signal, Non-Tag-like Signal | Informative Signal | Environmental uncertainty | -- | B+B*C |
| Li et al [49], 2019 | -- | Third Party, Internal Party | Offline Signal, Online Signal | Tag-like Signal, Non-Tag-like Signal | Informative Signal | -- | -- | B+B*B |
| Shah et al [19], 2019 | -- | Third Party, Internal Party | Offline Signal, Online Signal | Tag-like Signal, Non-Tag-like Signal | Informative Signal, Affective Signal | Environmental uncertainty | -- | B+B*C |
| Khurana et al [6], 2019 | -- | Third Party, Internal Party | Offline Signal, Online Signal | Tag-like Signal, Non-Tag-like Signal | Informative Signal | -- | -- | B+B*B |
| Yang and Zhang [70], 2019 | -- | Internal Party | Online Signal | Tag-like Signal | Informative Signal | -- | -- | B |
| Shan, Wang [53], 2019 | -- | Third Party, Internal Party | Offline Signal, Online Signal | Tag-like Signal, Non-Tag-like Signal | Informative Signal, Affective Signal | -- | -- | B+B*B |
| Chen et al [3], 2020 | -- | Internal Party | Online Signal | Non-Tag-like Signal | Informative Signal, Affective Signal | -- | -- | B |
| Zhang et al [51], 2019 | -- | Third Party, Internal Party | Offline Signal, Online Signal | Tag-like Signal, Non-Tag-like Signal | Informative Signal | -- | -- | B+B*B |
| Yang et al [41], 2020 | -- | Third Party, Internal Party | Offline Signal, Online Signal | Tag-like Signal | Informative Signal | Environmental uncertainty | -- | B+B*B+B*C |
| Wang et al [40], 2020 | -- | Internal Party | Online Signal | Tag-like Signal | Informative Signal | Environmental uncertainty | -- | B+B*C |
| Saifee et al [59], 2020 | -- | Internal Party | Online Signal | Tag-like Signal, Non-Tag-like Signal | Informative Signal, Affective Signal | -- | Disease knowledge | B*D |
| Peng et al [26], 2020 | -- | Internal Party | Online Signal | Non-Tag-like Signal | Informative Signal, Affective Signal | Environmental consistency | -- | B+B*C |
| Shukla et al [60], 2021 | -- | Third Party | Online Signal | Tag-like Signal | Informative Signal | Environmental consistency | -- | B+B*C |
| Yang et al [20], 2021 | -- | Third Party, Internal Party | Online Signal | Tag-like Signal, Non-Tag-like Signal | Informative Signal | -- | -- | B+B*B |
| Shah et al [61], 2021 | -- | Internal Party | Online Signal | Non-Tag-like Signal | Informative Signal, Affective Signal | -- | -- | B |
| Shah et al [7], 2021 | -- | Third Party, Internal Party | Online Signal, Offline Signal | Tag-like Signal, Non-Tag-like Signal | Informative Signal | Environmental uncertainty | -- | B+B*C |
| Wu et al [71], 2021 | -- | Internal Party | Online Signal | Tag-like Signal | Informative Signal | Environmental uncertainty | -- | B+B*C |
| Chen et al [68], 2021 | -- | Third Party, Internal Party | Offline Signal, Online Signal | Tag-like Signal | Informative Signal | -- | -- | B+B*B |
| Gong et al [36], 2021 | -- | Third Party, Internal Party | Offline Signal, Online Signal | Tag-like Signal, Non-Tag-like Signal | Informative Signal | -- | -- | B |
| Li et al [95], 2021 | -- | Third Party, Internal Party | Online Signal | Tag-like Signal | Informative Signal | -- | Illness-related psychological traits | B+B*D |
| Manga et al [72], 2021 | -- | Internal Party | Online Signal | Non-Tag-like Signal | Informative Signal, Affective Signal | -- | -- | B |
| Qiao et al [58], 2021 | -- | Third Party, Internal Party | Offline Signal, Online Signal | Tag-like Signal, Non-Tag-like Signal | Informative Signal | -- | -- | B+B*B |
| Qin et al [55], 2022 | Competence, benevolence, integrity | Third Party, Internal Party | Offline Signal, Online Signal | Tag-like Signal, Non-Tag-like Signal | Informative Signal | Environmental uncertainty | -- | B+A+B*C |
| Zhou et al [2], 2022 | -- | Third Party, Internal Party | Offline Signal, Online Signal | Tag-like Signal, Non-Tag-like Signal | Informative Signal, Affective Signal | -- | -- | B+B*B |
| Ouyang et al [47], 2022 | -- | Third Party, Internal Party | Online Signal | Tag-like Signal, Non-Tag-like Signal | Informative Signal, Affective Signal | -- | -- | B+B*B |
| Ma et al [18], 2022 | -- | Third Party, Internal Party | Online Signal | Tag-like Signal, Non-Tag-like Signal | Informative Signal | -- | -- | B+B*B |
| Shah et al [48], 2022 | -- | Third Party, Internal Party | Online Signal | Tag-like Signal, Non-Tag-like Signal | Informative Signal, Affective Signal | Environmental uncertainty | -- | B+B*C |
| Huang et al [73], 2022 | -- | Third Party, Internal Party | Online Signal, Offline Signal | Tag-like Signal, Non-Tag-like Signal | Informative Signal | Environmental uncertainty | -- | B+B*C |
| Yin et al [66], 2022 | -- | Third Party, Internal Party | Online Signal | Tag-like Signal, Non-Tag-like Signal | Informative Signal | Environmental consistency | -- | B+B*C |
| Fang et al [64], 2022 | -- | Internal Party | Online Signal | Non-Tag-like Signal | Affective Signal | Environmental uncertainty, Environmental competition | -- | B+B*C |
| Liu et al [8], 2022 | -- | Third Party | Offline Signal Online Signal | Tag-like Signal, Non-Tag-like Signal | Informative Signal, Affective Signal | -- | -- | B |
| Liu et al [37], 2022 | Competence | Third Party, Internal Party | Offline Signal, Online Signal | Tag-like Signal | Informative Signal | -- | -- | B*A |
| Ouyang and Wang [52], 2022 | -- | Third Party | Offline Signal Online Signal | Tag-like Signal, Non-Tag-like Signal | Informative Signal, Affective Signal | -- | -- | B+B*B |
| Yan et al [38], 2022 | -- | Third Party, Internal Party | Online Signals | Tag-like Signal, Non-Tag-like Signal | Informative Signal | -- | -- | B |
| Chen and Walker [45], 2023 | -- | Third Party | Offline Signal, Online Signal | Tag-like Signal, Non-Tag-like Signal | Informative Signal | -- | Disease knowledge | B+B*D |
| Ouyang et al [57], 2022 | -- | Third Party | Offline Signal, Online Signal | Tag-like Signal | Informative Signal | -- | -- | B+B*B |
| Chang et al [39], 2022 | -- | Internal Party | Online Signal | Non-Tag-like Signal | Informative Signal | -- | -- | A |
| Cheng et al [74], 2022 | -- | Third Party，Internal Party | Online Signal | Non-Tag-like Signal，Tag-like Signal | Informative Signal | Environmental uncertainty | -- | B+B*B+B*C |
| Shah et al [63], 2022 | -- | Third Party, Internal Party | Online Signal | Tag-like Signal | Informative Signal | Environmental uncertainty | -- | B+B*C |
| Yin et al [14], 2022 | -- | Third party | Offline Signal, Online Signal | Tag-like Signal | Informative Signal | -- | -- | B+B*B |
| Tan et al [54], 2023 | -- | Internal Party | Online Signal | Non-Tag-like Signal | Informative Signal, Affective Signal | -- | -- | B+B*A |
| Liu et al [17], 2023 | -- | Third party, Internal Party | Offline Signal, Online Signal | Tag-like Signal, Non-Tag-like Signal | Informative Signal | -- | -- | B+B*B |
| Fan et al [13], 2023 | -- | Third Party, Internal Party | Offline Signal, Online Signal | Tag-like Signal, Non-Tag-like Signal | Informative Signal | -- | -- | B+B*B |
| Zhou et al [50], 2023 | -- | Third Party | Offline Signal | Tag-like Signal, Non-Tag-like Signal | Informative Signal, Affective Signal | Environmental competition | -- | B+B*C |
| Xia [44], 2023 | -- | Third Party, Internal Party | Online Signal | Tag-like Signal, Non-Tag-like Signal | Informative Signal | -- | Health information literacy | B+B*D |
| Zhao and Cao [75], 2023 | -- | Third Party | Offline Signal, Online Signal | Tag-like Signal, Non-Tag-like Signal | Informative Signal | -- | -- | B+B*B |
| Dhakate and Joshi [76], 2023 | -- | Third Party, Internal Party | Offline Signal, Online Signal | Tag-like Signal, Non-Tag-like Signal | Informative Signal, Affective Signal | -- | -- | B |
| Xue et al [65], 2022 | -- | Third Party, Internal Party | Offline Signal, Online Signal | Tag-like Signal, Non-Tag-like Signal | Informative Signal | Environmental consistency, Environmental norms | -- | B+B*C |
| Chen et al [77], 2024 | -- | Third Party, Internal Party | Online Signal | Tag-like Signal, Non-Tag-like Signal | Informative Signal, Affective Signal | -- | -- | B |
| Chen et al [1], 2025 | -- | Third Party, Internal Party | Offline Signal, Online Signal | Tag-like Signal, Non-Tag-like Signal | Informative Signal | -- | -- | B*B |
| Fan et al [78], 2023 | -- | Internal Party | Online Signal | Non-Tag-like Signal | Informative Signal | -- | -- | B |
| Lantzy and Anderson [79], 2019 | -- | Internal Party | Online Signal | Non-Tag-like Signal | Informative Signal | -- | -- | B |
| Manyang et al [80], 2023 | -- | Third Party | Online Signal | Tag-like Signal | Informative Signal | -- | -- | B |
| Chen and Lee [24], 2023 | -- | Internal Party | Online Signals | Non-Tag-like Signal | Informative Signal | Environmental uncertainty | Health information literacy | B+B*B+B*D+B*C |
| Yang et al [81], 2023 | -- | Internal Party | Online Signal | Non-Tag-like Signal | Informative Signal, Affective Signal | Environmental consistency | -- | B+B*C |
| Jing et al [82], 2024 | -- | Internal Party | Online Signal | Non-Tag-like Signal | Informative Signal, Affective Signal | -- | -- | B |
| Jiang et al [83], 2024 | -- | Third Party | Online Signal | Tag-like Signal | Informative Signal | -- | -- | B |
| Li et al [5],2025 | -- | Internal Party | Online Signal | Non-Tag-like Signal | Informative Signal, Affective Signal | -- | -- | B |
| Du et al [84], 2024 | -- | Third Party, Internal Party | Offline Signal, Online Signal | Tag-like Signal, Non-Tag-like Signal | Informative Signal | Environmental consistency | -- | B+B*C |
| Yu et al [85], 2024 | -- | Internal Party | Online Signal | Tag-like Signal, Non-Tag-like Signal | Informative Signal | -- | -- | B+B*B |
| Zhou et al [86], 2024 | -- | Internal Party | Online Signal | Non-Tag-like Signal | Informative Signal | -- | -- | B |
| Feng et al [87], 2024 | -- | Third Party, Internal Party | Offline Signal, Online Signal | Tag-like Signal, Non-Tag-like Signal | Informative Signal | -- | -- | B |
| Yang et al [88], 2024 | -- | Internal Party | Online Signal | Tag-like Signal | Informative Signal | -- | Illness-related psychological traits | B+B*D |
| Yan et al [89], 2024 | -- | Internal Party | Online Signal | Non-Tag-like Signal | Informative Signal, Affective Signal | -- | -- | B |
| Chen and Wu [90], 2024 | -- | Third Party, Internal Party | Online Signal | Non-Tag-like Signal | Informative Signal, Affective Signal | -- | -- | B+B*B |
| Wei et al [91], 2024 | -- | Internal Party | Online Signal | Non-Tag-like Signal | Informative Signal, Affective Signal | -- | -- | B+B*B |
| Jing et al [92], 2024 | -- | Internal Party | Online Signal | Non-Tag-like Signal | Informative Signal | -- | Illness-related psychological traits | B+B*D |
| Liu et al [93], 2024 | -- | Internal Party | Online Signal | Tag-like Signal, Non-Tag-like Signal | Informative Signal | -- | Illness-related psychological traits | B+B*D |
| Bai et al [94], 2024 | -- | Internal Party | Online Signal | Tag-like Signal, Non-Tag-like Signal | Informative Signal, Affective Signal | -- | -- | B |
